# Prevalence and risk factors for VRE colonisation in a tertiary hospital in Melbourne, Australia: a cross sectional study

**DOI:** 10.1186/2047-2994-1-31

**Published:** 2012-10-08

**Authors:** Surendra Karki, Leanne Houston, Gillian Land, Pauline Bass, Rosaleen Kehoe, Sue Borrell, Kerrie Watson, Denis Spelman, Jacqueline Kennon, Glenys Harrington, Allen C Cheng

**Affiliations:** 1 Department of Epidemiology and Preventive Medicine, Infectious Disease Epidemiology Unit, Monash University, Melbourne, Australia; 2 Infection Control, Eastern Health, Melbourne, Australia; 3 Infection Prevention and Healthcare Epidemiology Infectious Diseases and Microbiology Unit, Alfred Health, Melbourne, Australia; 4 Infection Control Consultancy, Melbourne, Australia

**Keywords:** VRE, Colonisation, Acquisition, Prevalence, Risk factors, Australia, *VanB*, Antibiotics

## Abstract

**Background:**

Vancomycin-resistant *Enterococcus* (VRE) has been established as a significant health-care associated problem since its first isolation in Australia in 1994. In this study, we measured the point prevalence and identified risk factors associated with *vanB* VRE colonisation in a tertiary care hospital in Melbourne, Australia where VRE has been endemic for 15 years.

**Methods:**

A hospital-wide point prevalence survey was conducted on October 13, 2008 with colonisation detected using rectal swab culture. Patient’s demographic and medical information was collected through a review of medical records. Factors associated with VRE colonisation in univariate analysis were included in multivariate logistic regression model to adjust for confounding.

**Results:**

The prevalence of VRE colonisation on the day of screening was 17.5% (95% CI, 13.7 to 21.9). VRE was detected from patients in each ward with the prevalence ranging from 3% to 29%. Univariate analysis showed the use of any antibiotic, meropenem, ciprofloxacin, diarrhoea and longer length of hospital stay were associated with increased risk of VRE colonisation (p<0.05). However, age, sex, proximity to VRE positive cases, use of other antibiotics including cephalosporins, vancomycin were not associated with increased risk (P>0.05). Multivariate analysis showed the exposure to meropenem (p=0.004), age (≥65 years) (p=0.036) and length of stay ≥7 days (p<0.001) as independent predictors of VRE colonisation.

**Conclusion:**

Our study suggests that exposure to antibiotics may have been more important than recent cross transmission for a high prevalence of *vanB* VRE colonisation at our hospital.

## Background

The emergence and spread of vancomycin-resistant *Enterococcus* (VRE) in health care settings has added risks and complexities in patient management. VRE can cause a variety of health care-associated infections, particularly bacteraemia and urinary tract infections. Most enterococcal infections are caused by two species, *E. faecalis* or *E. faecium*. VRE was first reported in Europe in 1986 and subsequently worldwide [1]. In Australia, the first case of VRE was reported in 1994 and by 1998 from all other major cities [2,3]. The predominant VRE genotype circulating in Australia is *E. faecium vanB*, in contrast to the *vanA* genotype which is predominant in the United States and Europe [3,4]. Recent surveys have shown an increase in the prevalence of vancomycin resistance in clinical isolates of enterococci in Australia [5]. A VRE prevalence as high as 19.1% was reported during an outbreak among hospital inpatients in Victoria [6]. Faecal colonisation with VRE in the community has been demonstrated in Australia but appears to be rare (prevalence 0.2%) although the *vanB* gene appears to be common in commensal bacteria including *Clostridium* spp [7-9].

Previous studies have identified various risk factors for VRE colonisation including advanced age, severe underlying illness, inter-hospital transfer, nursing home residency, extended hospitalization, specialized nutritional support, central venous catheterization, haematologic malignant tumours, solid organ allograft, chronic haemodialysis, antibiotic exposure to vancomycin, third-generation cephalosporins, metronidazole, antibiotics with anti-anaerobic activity, exposure to multiple antibiotics and prolonged duration of antibiotic therapy [10]. However, most of those studies involve high risk patients during outbreaks associated with *vanA* genotype. A previous study in Melbourne hospitals in 1998 identified broad spectrum antibiotics as significant risk factor for new colonisation with *vanB* VRE [11].

VRE colonisation can lead to infections which prolong hospital stay, increase the cost of care and increase morbidity and mortality [12,13]. The identification of modifiable risk factors may assist in identifying targets for intervention to reduce the incidence of VRE colonisation. In this study, we measured the prevalence of VRE colonisation in a hospital-wide point prevalence survey. We examined downstream risk factors for colonisation with implications for VRE control policies, including antibiotic exposure and patient placement, in a tertiary referral hospital in Melbourne, Australia where *vanB* VRE has been endemic for many years.

## Methods

### Setting and study design

The Alfred hospital is a major tertiary teaching hospital in Melbourne, Australia with 427 beds. The hospital provides general services as well as broad range of specialist care including referral services for trauma, cystic fibrosis and heart/lung transplantation, HIV/AIDS, bone marrow transplantation as well as specialist intensive care facilities.

We conducted a point prevalence survey on October 13, 2008 at the Alfred Hospital. All patients who were present in the hospital (including inpatients, patients in the emergency department, and day surgery but excluding psychiatry) at 8.00 a.m. were approached to participate. Patients were provided with information sheets (including advice about the potential consequences of VRE colonisation) and verbal consent was obtained for participation. Patients were included irrespective of known VRE status. Patients were excluded if they were not able to consent, were for palliative care only, had been discharged prior to being swabbed or they declined to participate. Single rectal swabs were primarily taken by nursing staff. Clinical data collection included demographic details, bed location of all patients, antibiotic use (at least 12 hours before the collection of rectal swab in current admission), ICU exposure, and previous hospital admissions in patients that were found to be positive for VRE.

At the time of the survey, our VRE policy recommended the screening of all ward patients where a new case of VRE infection or colonisation was detected. There were no systemic screening practices for patients in high risk areas or testing of diarrhoeal stools prior to this study. Patients with known VRE colonisation or infection were isolated in single rooms with a dedicated toilet if they had faecal incontinence or diarrhoea. Healthcare workers were required to use gloves when entering the room, and gloves and gown if contact with body fluids was anticipated. Hospital floors were cleaned daily with 1000 part per million (ppm) sodium hypochlorite.

A nested case control (1:2) study was conducted to identify risk factors associated with VRE colonisation. A case was defined as a patient confirmed to be positive by standard microbiological methods for VRE isolated from rectal swabs taken for the point prevalence survey. A control was defined as a patient confirmed to be negative for VRE colonisation on the same survey. For each case, two unmatched controls were selected at random from eligible patients.

### Microbiology

Rectal swabs were taken either by the patients themselves following instruction, or by nursing staff. The swabs were then plated onto bile aesculin media (BBL Enterococcosel agar, Cockeysville, MD) with vancomycin 6 μg/mL and incubated at 37°C for up to 72 hours. Enterococci species were identified using the VITEK-2 system (bioMérieux). Polymerase chain reaction was used to detect the *vanA* or *vanB* genes as described previously. [14] VRE colonisation was defined if an isolate of *E. faecalis* or *E. faecium* with *vanA* or *vanB* gene was detected. Ribotyping of the isolates was performed using the Riboprinter Microbial Characterization system as previously described [15,16].

### Statistical methods

Standard statistical methods were used to summarize categorical and continuous measures and to compare proportions. Univariate analysis was performed to calculate unadjusted odds ratio. All variables with a p-value of <0.2 in univariate analysis were included in the multiple logistic regression model to examine independently associated risk factors for VRE colonisation. The association was considered statistically significant if p<0.05. Statistical analyses were performed using Stata statistical software (Version 10; Stata Corp, College Station, Texas).

### Ethics

As this VRE survey represented a quality assurance activity, we did not obtain a formal ethical approval to perform the point prevalence survey. However, we counselled patients about the significance of VRE and provided written information, and obtained verbal consent to take rectal swabs from all patients and/or their relatives. We obtained approval from the Alfred Health Human Research Ethics Committee and Monash University Human Research Ethics Committee to retrieve demographic and clinical information from the medical records of patients for the case control study.

## Results

A total of 434 patients were present in hospital at the time of survey. Among the total, 331 (77%) were screened for VRE colonisation; 103 patients (23%) could not be screened either due to refusal, discharge from hospital prior to the time of swabbing, patients not being present in the ward at the time of screening, or if the patient was for palliative care.

The prevalence of VRE colonisation on the day of screening was 58 of 331 patients (17.5%, 95% CI, 13.7% to 21.9%). The proportion of patients colonised with VRE was 3% in the emergency department and ranged from 8% to 29% in inpatient wards. Of the 58 VRE isolates, 57 were found to be *Enterococcus faecium* and one was *Enterococcus faecalis*. All the isolates were positive for *vanB* resistance genotype and no *vanA* resistance genotype was detected. All together, 9 ribotypes were detected with the majority of isolates belonging to either of the two ribotypes (S-7, n=25, 43%; S-5, n=23, 39%). However, more than 1 ribotype was present in all wards where there was more than one VRE-colonised patient present.

Of the total 58 patients found to be VRE colonised, 46 (79.3%) were not previously known to be VRE colonised and 12 (20.7%) were known to be previously colonised. A further 13 patients had a history of VRE colonisation but VRE was not detected on screening at the time of the survey.

### Risk factors for VRE colonisation

We compared the exposure to probable risk factors in 58 cases and 116 controls.

Significantly higher proportions of cases had prior exposure to one or more antibiotics (odds ratio (OR) 3.83, 95% CI 1.79 to 8.54). Similarly, cases had a longer length of hospital stay compared to controls (P<0.001). Patients with VRE colonisation were more likely to have been admitted for ≥7 days (OR 4.86, 95% CI 2.30 to 10.51). Univariate analyses identified prior exposure to any antibiotic (p <0.001), exposure to meropenem (p=0.001), ciprofloxacin (p=0.03), diarrhoea (p=0.03) as associated with the detection of VRE colonisation (Table 1). However, the prior use of vancomycin (p=0.33), ticarcillin-clavulanate (p=0.11), pipericillin-tazobactam (p=0.07), metronidazole (p=0.16) and any cephalosporins were not associated with colonisation status. Proximity to other VRE positive cases either in same side of the ward or located within next 2 rooms was not associated with VRE colonisation (p>0.05) (Table 1).

**Table 1:** Factors associated with VRE detection in cases and controls (Univariate analysis)

**Variables**	**Cases**	**Controls**	**Unadjusted OR**	**P value**
Participants	58	116	-	-
Age ≥ 65 (years)	35 (60.3%)	56 (48.2%)	1.63 (0.82 to 3.26)	0.13
Male	36 (62.1%)	74 (63.8%)	0.92 (0.46 to 1.88)	0.82
*Antibiotics*				
Exposure to any antibiotic	45 (77.6%)	55 (47.4%)	3.83 (1.79 to 8.54)	<0.001
Meropenem	8 (13.8%)	2 (1.7%)	9.12 (1.71 to 89.92)	0.001
Antibiotics other than meropenem	39 (67.2%)	54 (46.5%)	4.5 (2.06 to 10.71)	0.001
Vancomycin	13 (22.4%)	19 (16.38%)	1.47 (0.61 to 3.46)	0.33
Teicoplanin	2 (3.4%)	2 (1.7%)	2.03 (0.14 to 28.63)	0.47
Any cephalosporin	19 (32.8%)	32 (27.6%)	1.27 (0.60 to 2.66)	0.47
Ceftriaxone	11 (18.9%)	24 (20.7%)	0.89 (0.36 to 2.11)	0.78
Cefotaxime	2 (3.4%)	0	-	-
Ceftazidime	5 (8.6%)	4 (3.4%)	2.64 (0.54 to 13.79)	0.14
Cefepime	1 (1.7%)	6 (5.2%)	0.32 (0.01 to 2.76)	0.27
Metronidazole	5 (8.6%)	19 (16.4%)	0.48 (0.13 to 1.43)	0.16
Ciprofloxacin	11 (18.9%)	9 (7.8%)	2.78 (0.96 to 8.11)	0.03
Ticarcillin-clavulanate	11 (18.9%)	12 (10.3%)	2.02 (0.74 to 5.41)	0.11
Ampicillin	1 (1.7%)	3 (2.6%)	0.66 (0.01 to 8.45)	0.72
Gentamicin	2 (3.4%)	9 (7.8%)	0.42 (0.04 to 2.16)	0.27
Piperacillin-tazobactam	5 (8.6%)	3 (2.6%)	3.55 (0.65 to 23.53)	0.07
*Other factors*				
ICU admission	18 (31.1%)	26 (22.4%)	1.55 (0.71 to 3.33)	0.21
Within 2 rooms of positive case	30 (62.5%)	54 (65.8%)	0.86 (0.38 to 1.94)	0.69
On same side as positive case	48 (82.8%)	83 (77.6%)	1.38 (0.57 to 3.53)	0.43
Diarrhoea	12 (21.1%)	11 (9.5%)	2.54 (0.94 to 6.85)	0.03
Length of stay (Median days)	11.5 (7–30)	6.0 (3–13)	-	<0.001
Length of stay ≥7 days	43 (74.2%)	43 (37.1%)	4.86 (2.30 to 10.51)	<0.001

In a multivariate logistic regression model, exposure to meropenem (adjusted OR; 12.24, 95% CI 2.24 to 66.77), age ≥ 65years (adjusted OR 2.19, 95% CI 1.05 to 4.58) and length of stay ≥7 days (adjusted OR 4.69, 95% CI 2.25 to 9.73) were independently associated with VRE colonisation (Table 2). After adjusting for age ≥ 65years, length of stay ≥7 days and exposure to meropenem, exposure to any antibiotic other than meropenem (compared to patients that did not receive any antibiotics) was independently associated with VRE colonisation (adjusted OR 2.95, 95% CI 1.27 to 6.88). In total, 16 (9.2%) patients in case–control study were from emergency department and short stay units. A sensitivity analysis excluding the patients in these units did not alter any of the above effects, significantly.

**Table 2:** Factors associated with VRE detection in cases and controls (multivariate analysis)

**Risk factor**	**Adjusted OR (95% CI)**	**p value**
Meropenem	12.24 (2.24 to 66.77)	0.004
Age (≥65 years)	2.19 (1.05 to 4.58)	0.036
Length of stay ≥7 days	4.69 (2.25 to 9.73)	<0.001

## Discussion

We conducted a hospital-wide point prevalence survey to measure the prevalence of VRE, as our previous surveillance activities were targeted to patients felt to be at risk and therefore may have resulted in selection biases. In this survey, we confirmed that *vanB* VRE is endemic in inpatients at our hospital. We found a lower prevalence in short stay areas, such as the emergency department (3%) and the short stay unit, but prevalence was 8-29% in other inpatient areas. This is higher than reported by previous studies conducted at Melbourne hospitals [11,17-19]. This is also in contrast to previous studies demonstrating that VRE colonisation was restricted to particular high risk inpatient areas [11]. Our finding that 80% of the VRE colonised patients in the survey were newly detected cases demonstrates that the previous screening strategy of surveying patients only on wards when a new clinical isolate of VRE was detected is inadequate.

Prima facie, our results suggest that antibiotic selection pressures appear to play a larger role in determining VRE colonisation than cross-transmission at our institution. Exposure to antibiotics, particularly meropenem, was strongly associated with VRE colonisation; this is consistent with previous studies suggesting that antibiotic regimens with activity against anaerobic bacteria are potent risk factors in the development of VRE [20-22]. The *vanB* gene is known to be present in commensal enteric bacteria such as *Clostridium*, and transmission of this gene to *E. faecium* has been demonstrated *in vivo*[23]. In other studies, a variety of other antibiotics have been implicated as risk factors for VRE colonisation, including vancomycin, metronidazole, piperacillin-tazobactam, ticarcillin-clavulanate and third generation cephalosporins [24-26]. However, our analysis comparing meropenem and non-meropenem antibiotic use with no antibiotic use showed the exposure to antibiotics other than meropenem is also associated with VRE colonisation. Thus, our study may have been underpowered to detect differences at the level of individual antibiotics other than meropenem.

We found ambiguous evidence regarding the extent of cross transmission of VRE, which has been shown to be important in previous studies [27,28]. We did not find proximity to other VRE colonised patients to be a risk factor for VRE detection. However, this does not exclude the possibility of cross transmission contributing to endemicity, as transmission may have occurred prior to the time of the survey and would not be detected with frequent patient movements. Conversely, the finding that ribotyping only demonstrated two large clones of VRE in patients does not necessarily implicate extensive cross transmission. If transmission was significant, we would expect a lower diversity among individual wards; however, we found that in all wards with at least two VRE colonised patients had at least two ribotypes. We did not have access to pulsed field gel electrophoresis, regarded as the gold standard for epidemiological investigations of VRE [29].

The purpose of this study was to examine “downstream” exposures that had implications for VRE control policies, rather than patient-specific “upstream” risk factors that are not modifiable. However, we did find older age and longer length of stay in hospital as independent predictor of VRE colonisation. This is reflected in the low colonisation rate in patients with a short length of stay, including emergency and short stay units.

A strength of this study is that we conducted a hospital-wide survey including a broad range of patients, thus minimizing selection biases [30]. A limitation of this cross-sectional study is that we were not able to determine the timing of acquisition of VRE in the patients identified as being colonised; therefore, patients may have acquired VRE in the remote past, with antibiotic use merely amplifying existing colonisation. In addition, we do not have data on patient co-morbidities, previous invasive procedures, and indwelling devices, which may have confounded our findings. We chose to perform rectal swabs due to practical reasons but previous studies have demonstrated that the sensitivity of these specimens is around 79%, we might have missed detection of few patients with low VRE density [31,32]. We did not perform separate broth enrichment before the agar plate culture, which may lead to an underestimation of the true prevalence [33]. This may have resulted in misclassification bias in assigning VRE colonised patients as controls and bias the case control study towards the null hypothesis. As the sensitivity of VRE detection is likely to be related to bacterial density, it is likely that detection of VRE in screening is the product of two processes, that of new acquisition of VRE (whether by endogenous generation or exogenous exposure) and amplification of existing low level colonisation by antibiotics [34].

## Conclusions

The endemic and high prevalence of *vanB* VRE in our setting may have been maintained by higher exposure to antibiotics. Although cross transmission remains a possibility and is supported by predominance of two large clones of VRE, colonisation status did not appear to be associated with proximity to other colonised patients. Our data suggest that antibiotic stewardship efforts may be more effective in reducing the spread of VRE in our hospital.

## Competing interests

The authors declare that they have no competing interests.

## Authors’ contributions

The point prevalence study was designed by GH, LH, GL, PB, SB, RK, DS. The study was primarily co-ordinated by LH with support from GH, LH, GL, PB, SB, RK, including identifying patients, co-ordinating swab collection and collecting data on cases. DS oversaw microbiological testing and typing. LH and KW managed the data and performed the initial analysis of cases. AC and SK designed and analysed the case control study with assistance from JK and DS. SK collected the data on controls and drafted the manuscript. All authors interpreted the results and revised the manuscript.
